# ECNR European Congress of NeuroRehabilitation 2019

**DOI:** 10.25122/jml-2019-1009

**Published:** 2019

**Authors:** Victor Lorin Purcarea

**European Congress of NeuroRehabilitation 2019** was held between 9 and 12 October in Budapest and benefited from participation of many neurologists, neurosurgeons, IT specialists, physiotherapists, occupational therapists, kinesiotherapists, logopedists, psychologists, medical assistants, psychiatrists, both from Romania and from abroad.

The central theme was „***Impairment, disability, handicap: So little done, so much to do***”.

**Figure 1:**
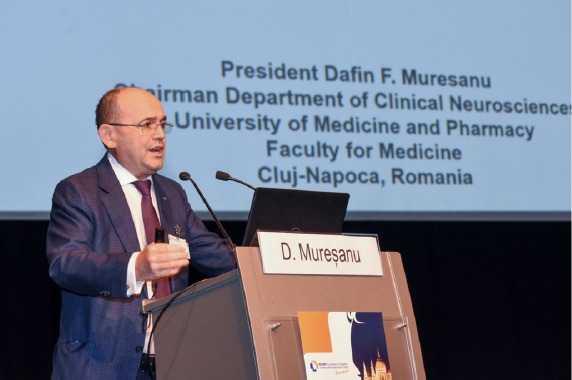
Prof. Dafin Muresanu, MD, PhD, President of European Congress of NeuroRehabilitation

**Figure 2:**
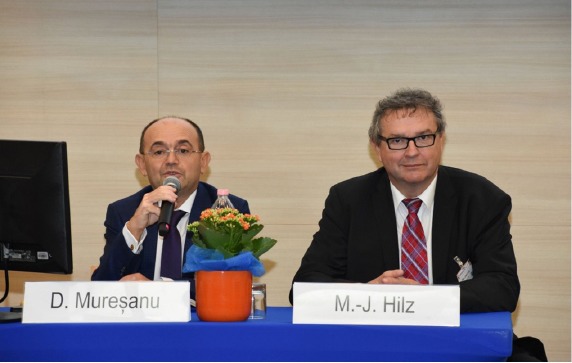
Prof. Dafin Muresanu, MD, PhD – World Stroke Organization, Romanian Society for NeuroRehabilitation, Cluj-Napoca, Romania, and M.-J. Hilz - European Academy for Neurology, Erlangen, Germany

**Figure 3:**
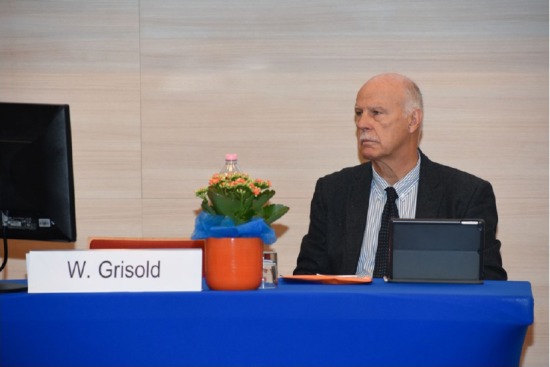
W. Grisold – World Federation of Neurology, Vienna, Austria

**Figure 4:**
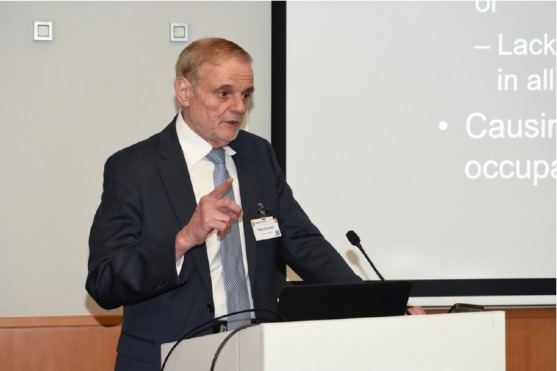
Natan Bornstein – World Stroke Organization, Tel Aviv-Yafo, Israel

**Figure 5:**
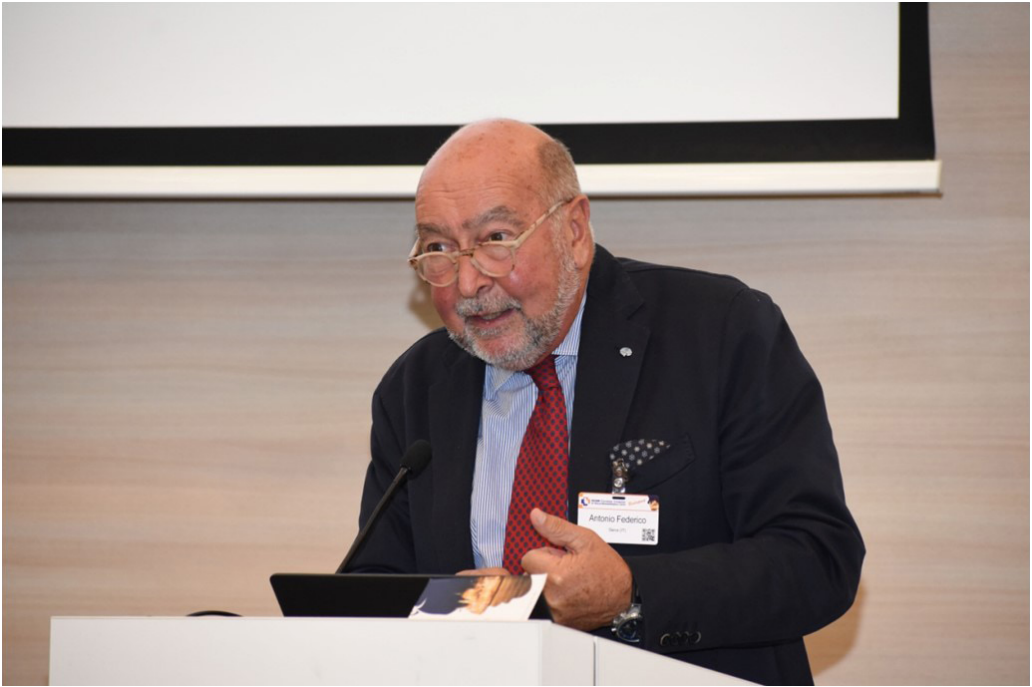
Antonio Federico – European Academy for Neurology, Siena, Italy

Similar to previous editions, the congress has maintained its international character, providing experts with a platform in all areas related to neurology and rehabilitation and hosting over 60 sessions with many international speakers (Figures 1-5).

The Organizing Committee of the event included personalities such as: Dafin Muresanu – President, Volker Homberg – Program President, Leopold Saltuari, Giorgio Sandrini, Heinrich Biner and Karin Diserens.

The event was organized in collaboration with the Bulgarian Society of NeuroRehabilitation, European Academy for Neurology, Romanian Society for Neuropsychological and Motor Rehabilitation, Romanian Society for NeuroRehabilitation, etc.

In the opening of the congress, Prof. Dafin Muresanu, MD, PhD, approached the subject of changing the philosophy in neurorehabilitation, referring to the three basic pillars: **theory and research, evidence based on effectiveness and safety, international validation of the obtained results**. He also mentioned the need to develop guides in partnership with the societies in the field and to implement them in the EFNR member societies.

The aim of the congress was to keep the dialogue open between the countries of Europe but also with colleagues in the field, from all over the world.

As it is well known, the goal of all neurologists is continuous research, collaboration in order to avoid damage and disability because of brain and cardiovascular diseases, accidents and injuries or functional disorders.

Among the novelties of the event were the following: interactive teaching day dedicated to young researchers who took part in the congress; „Ask the expert!” breakfast sessions, in which 10 professors offered their experience, advice and knowledge in two parallel 60-minutes morning sessions; career development, which implied the improvement of knowledge on how to organize a study, how to best write an academic paper, and also how to choose the career path; young experts’ battle, in which young specialists, aged below 35 years, participated in debates that also involved the audience; best oral presentations, implied the presentation of the six best rated abstracts that were presented in 30-minutes lectures.

Moreover, among the key topics of the congress were the following: “Impairment vs. compensation oriented approaches in stroke – when to switch”, “New vistas for education in neurorehabilitation in Europe”, “The enigma of “proportional recovery” – impact for neurorehabilitation”, “Innovative strategies for neurorehabilitation trials”, “The rationales of cognitive training strategies”.

